# An Analysis of the Extent of Social Inclusion and Equity Consideration in Malawi’s National HIV and AIDS Policy Review Process

**DOI:** 10.15171/ijhpm.2017.87

**Published:** 2017-07-29

**Authors:** Mathews Junior Chinyama, Malcolm MacLachlan, Joanne McVeigh, Tessy Huss, Sylvester Gawamadzi

**Affiliations:** ^1^Centre for Global Health, Trinity College Dublin, Dublin, Ireland.; ^2^Department of Nutrition, HIV and AIDS, Lilongwe, Malawi.; ^3^Centre for Global Health & School of Psychology, Trinity College Dublin, Dublin, Ireland.; ^4^Centre for Rehabilitation Studies, Stellenbosch University, Stellenbosch, South Africa.; ^5^Olomouc University Social Health Institute, Palacky University, Olomouc, Czech Republic.

**Keywords:** EquiFrame, EquIPP, Equity, Social Inclusion, Vulnerable Groups, Human Rights Core Concepts

## Abstract

**Background:** Equity and social inclusion for vulnerable groups in policy development processes and resulting documents remain a challenge globally. Most often, the marginalization of vulnerable groups is overlooked in both the planning and practice of health service delivery. Such marginalization may occur because authorities deem the targeting of those who already have better access to healthcare a cheaper and easier way to achieve short-term health gains. The Government of Malawi wishes to achieve an equitable and inclusive HIV and AIDS Policy. The aim of this study is to assess the extent to which the Malawi Policy review process addressed regional and international health priorities of equity and social inclusion for vulnerable groups in the policy content and policy revision process.

**Methods:** This research design comprised two phases. First, the content of the Malawi HIV and AIDS Policy was assessed using EquiFrame regarding its coverage of 21 Core Concepts of human rights and inclusion of 12 Vulnerable Groups. Second, the engagement of vulnerable groups in the policy process was assessed using the EquIPP matrix. For the latter, 10 interviews were conducted with a purposive sample of representatives of public sector, civil society organizations and development partners who participated in the policy revision process. Data was also collected from documented information of the policy processes.

**Results:** Our analyses indicated that the Malawi HIV and AIDS Policy had a relatively high coverage of Core Concepts of human rights and Vulnerable Groups; although with some notable omissions. The analyses also found that reasonable steps were taken to engage and promote participation of vulnerable groups in the planning, development, implementation, monitoring and evaluation processes of the HIV and AIDS Policy, although again, with some notable exceptions. This is the first study to use both EquiFrame and EquIPP as complimentary tools to assess the content and process of policy.

**Conclusion:** While the findings indicate inclusive processes, commitment to Core Concepts of human rights and inclusion of Vulnerable Groups in relation to the Malawi HIV and AIDS Policy, the results also point to areas in which social inclusion and equity could be further strengthened.

## Background

The Alma-Ata declaration of 1978^1^ brought to the attention of all countries that attaining ‘Health for All’ begins with primary healthcare, and that the inclusion of every citizen in health services is imperative. Primary healthcare methods must be acceptable to service-users, while making technology universally accessible to individuals and families in the community through their full participation and at a cost that the community and the country can afford.^1,2^ The declaration continues to guide countries in the formulation of policies that aim at achieving ‘Health for All.’

Nevertheless, even though the Alma-Ata declaration has been in existence for almost four decades, health for all remains a challenge. Today the world continues to face the challenge of ensuring access to healthcare in an effort to improve the quality of life for the 36.7 million people globally, currently living with the HIV virus. In 2015, 2.1 million people were diagnosed with new HIV infections. Of these, 19 million people were living in eastern and southern Africa, with women accounting for more than half of the total number of people living with HIV in this region.^3^ Sub-Saharan Africa has the biggest burden of HIV and AIDS compared to other regions of the world. For example, in Eastern Europe and central Asia, there were approximately 1.5 million people living with HIV in 2015, with 190 000 new HIV infections in the region.^3^ Importantly, HIV has disproportionately affected the world’s poorest and most marginalized people.^4^

Equity is an ethical norm that is closely linked to human rights. Equity and human rights principles aim to achieve equal opportunities in health for everyone, especially for vulnerable groups who may be discriminated against or marginalized socially.^5,6^ The promotion and protection of human rights is therefore essential for expanding access to health services, especially for those who are most vulnerable. Of particular relevance within the Malawi policy context is society’s treatment of two such groups: men who have sex with men (MSM) and people with disabilities. We will discuss this in further detail later in the article. The Global Fund, with its mandate to direct resources to support the fight against HIV, TB and Malaria, included an explicit human rights objective in its strategy and this has led to efforts by governments to integrate human rights in their development strategies and address human rights-related barriers.^7^

Realization of equity, through consideration and inclusion of vulnerable groups in all aspects of life, is one of the central objectives of development agenda.^8^ As observed by Tamburlin,^9^ the current global economic trends scientific and technological developments, may all in fact contribute to disparities in vulnerability, differential-exposure to risk factors for ill health, and unequal access to health services leading to governments not achieving the agenda. Governments thus face considerable challenges in realizing equity in health.^9^

The marginalization of vulnerable groups is often overlooked in both the planning and practice of health services. Such marginalization may occur because authorities deem targeting of those who already have better access to healthcare a cheaper and easier way to achieve short-term health gains.^10^ Yet addressing the needs of vulnerable groups is central to addressing the HIV and AIDS pandemic,^8^ and efforts may be hampered by the exclusion of such groups from prevention and treatment programmes. HIV-related human rights violations, especially stigma and discrimination, gender inequality, and violence against women and girls, increase the risk of HIV infection.^11^ Stigma and discrimination may prevent people from accessing HIV prevention, treatment, care and support services.

The 2030 development agenda and the Sustainable Development Goals (SDGs) have placed much greater emphasis on the importance of equity and inclusion in human development. For example, Goal 16 is *“to promote peaceful and inclusive societies for sustainable development, provide access to justice for all and build effective, accountable and inclusive institutions at all levels.”*^12^ However, this ambitious goal can be achieved only if countries and institutions include vulnerable groups in policy processes. Solar and Irwin^13^ argue that inclusion of vulnerable groups in policy processes ensures that the interests and needs of the most marginalized in society are represented. Inclusion of vulnerable groups in policy processes allows such groups to have a degree of input and control over such processes. This provides a further avenue for vulnerable groups and the citizenry to hold their government accountable.^14^ In other words, inclusion of vulnerable groups in policy processes should be translated into the changes they are seeking. Active participation of vulnerable groups in policy processes supports the operationalization and implementation of the developed policy.^15^

Of note, participation in policy processes is not a privilege but a human right held by every individual, which extends beyond the policy formulation stage.^16^ Participation is a procedural right and it is underpinned by a number of civil and political rights such as access to information, as well as freedom of speech and of assembly. People should be included and allowed to participate in the implementation, monitoring and evaluation of policies that affect them.^17^ Therefore, governments should create forums that are both deliberative and participatory to complement existing structures that represent vulnerable groups and directly involve all marginalized groups.^13,18^ In order for vulnerable groups to contribute effectively, they should be allowed to participate in policy processes in a setting where they will be free to do so without undue outside influence.

Importantly, an inclusive policy process does not always lead to an equitable and inclusive policy outcome. The analysis of sexual and reproductive health policies in Ukraine, Scotland, Moldova and Spain^19^ revealed that successful promotion of the inclusion of vulnerable groups and core concepts of human rights in policy documents does not guarantee the automatic and accurate reflection of the needs and demands of those vulnerable groups.^20^ Indeed, participation in policy processes can be used as a signature of authentication, while not ensuring that the actual content of policy revision reflects the shifts that participants sought. It is therefore important to try to combine an analysis of policy process and policy content.

The government of Malawi is now seeking to target those most at risk of becoming infected with HIV or of infecting others, with the aim of targeting interventions in those settings where most HIV-affected individuals are living. The government is therefore focused on reaching out to neglected settings, key populations, such as MSM and female sex workers, and other vulnerable populations, including prisoners, estate workers and mobile groups of people. However, the government also recognizes that the HIV epidemic cannot be effectively addressed if human rights and gender issues are not respected.^21^

Despite the efforts the country has taken to fight HIV through different strategic documents, still it is not clear whether these documents really address the needs of the most marginalized members of society. It is also not clear whether the vulnerable groups who are the primary beneficiaries of these documents are included in the development process. Recently, MacLachlan et al,^8^ conducted an analysis on health policies from Malawi, Namibia, South Africa and Sudan to establish the extent to which these documents promoted universal, equitable and accessible health services. The study revealed that Malawi’s HIV policy had moderate coverage of Vulnerable Groups in relation to Core Concepts of human rights. However, a great deal of literature suggests that vulnerable groups are critical to addressing the HIV and AIDS pandemic.^10^ Therefore, it is of importance to assess how the policy was developed, and the degree to which vulnerable groups were part of this process. The aim of the present study therefore is to assess the extent to which the Malawi National HIV and AIDS Policy review process addressed regional and international health priorities of social inclusion and equity of vulnerable groups in the policy content and in the policy revision process.

## Methods

The research design was exploratory in nature. It consisted of reviewing and analyzing the Malawi National HIV and AIDS Policy. The analysis was performed in two phases: the first analysis was conducted on the content of the policy in relation to coverage of Core Concepts of human rights and inclusion of Vulnerable Groups. This analysis was performed using EquiFrame.^22^ The second analysis involved assessing the policy review process that was conducted in relation to vulnerable groups’ engagement and participation in the review process. The engagement of vulnerable groups was assessed and analyzed using EquIPP, a tool designed to complement EquiFrame. The EquIPP tool supports equity and inclusion in the process of policy development, implementation and evaluation.^23^

### EquiFrame Analysis

EquiFrame is a tool developed to provide a standardized policy analysis tool when developing and analyzing public policies within a human rights framework.^22^ EquiFrame lists 21 Core Concepts of human rights (Appendix 1) as well as 12 Vulnerable Groups (Appendix 2).

#### Scoring

Each Core Concept received a score ranging from 1 to 4. This was a rating of the quality of commitment to the core concept within the policy.^22^ Core Concepts were scored as follows:

0 = If the Concept was not mentioned at all.
1 = If the Concept was only mentioned.
2 = If the Concept was mentioned and explained.
3 = If specific policy actions were identified to address the Concept.
4 = If an intention to monitor the Concept was expressed.

If any of the 21 Core Concepts were seemingly not relevant to the context of the policy, they were scored as not applicable.

#### Summary of the EquiFrame Indices

The EquiFrame indices are summarized below:

1. *Core Concept Coverage:* The policy was examined with respect to the number of Core Concepts mentioned out of the 21 Core Concepts identified. This ratio was expressed as a rounded-up percentage. Additionally, the actual terminologies that were used to define the Core Concepts were extracted to allow for future qualitative analysis and cross-checking between raters.
2. *Vulnerable Group Coverage:* The policy was examined with respect to the number of Vulnerable Groups mentioned out of the 12 Vulnerable Groups identified. This ratio was expressed as a rounded-up percentage. Additionally, the actual terminologies that were used to define the Vulnerable Groups were extracted to allow for future qualitative analysis and cross-checking between raters.
3. *Core Concept Quality:* The policy was examined with respect to the number of Core Concepts within it that were rated as 3 or 4 (as either stating a specific policy action or intention to monitor that action) out of the 21 Core Concepts identified. When several references to a Core Concept were found to be present, the top quality score received was recorded as the final quality scoring for the respective Core Concept.
4. *Overall Summary Ranking:* The policy was given an Overall Summary Ranking in terms of it being of ‘High,’ ‘Moderate’ or ‘Low’ standing based on the following criteria:

(*i*) ‘High’ if the policy achieved ≥50% on all of the three scores above.
(*ii*) ‘Moderate’ if the policy achieved ≥50% on two of the three scores above.
(*iii*) ‘Low’ if the policy achieved <50% on two or three of the three scores above.

### EquIPP Analysis

EquIPP is a framework that promotes greater equity and social inclusion in policy processes. The framework strives to create an inclusive policy process for vulnerable groups who are frequently sidelined and marginalized in policy processes. EquIPP aims to provide vulnerable groups with the opportunity to ensure that their interests and concerns are adequately represented throughout policy development or revision processes. The tool assesses what happens before the production of the policy and what happens once it has been produced.^23^ EquIPP has 17 Key Actions, which form a guide to an equitable and inclusive policy process. The Key Actions focus on formulation, planning, operationalization and budgeting, implementation, monitoring and evaluation, dissemination, and feedback loops of policies.^23^

The EquIPP analysis was conducted in Lilongwe, Malawi, targeting individuals who were involved in the revision process of the policy. The respondents comprised the following clusters of the Malawi National HIV and AIDS Response: Public Sector, Civil Society, Private Sector and Development Partners (Appendix 3). The CSOs in particular included the Centre for the Development of People, an organization that promotes the rights of sexual minority groups (sex workers, lesbian, gay, bisexual, and transgender [LGBT], prisoners). Respondents were recruited through an Expert Purposive sampling method. The Department of Nutrition, HIV and AIDS provided the list of participants from which the respondents were sampled. In total, 10 interviews were conducted. Data collection and analysis also included documented information (Appendix 4).

#### Scoring

The EquIPP tool uses an assessment matrix or checklist of the extent to which a policy qualifies as equitable and inclusive. During the analysis, each Key Action received a score of 0 to 7. The score range indicated the commitment to equity and inclusion in each individual Key Action.^23^ Each Key Action received a score of:

0 = Absent: If there was no evidence it had been considered.
1 = Recognition: If there was evidence of awareness but no associated action.
2 = Minor action: If there was evidence of token or minimal efforts to engage.
3 = Moderate action: If there was evidence of clear but incomplete or partial engagement.
4 = Comprehensive action: If there was evidence that all reasonable steps to engage were taken.
5 = Policy Evaluation: If there was reference to Key Action in core documents.
6 = Process Evaluation: If there was evidence gathered from diverse stakeholders of satisfaction with the process of engagement.
7 = Outcome Evaluation: If there was evidence gathered from diverse stakeholders of satisfaction with the outcomes of engagement.

For the purpose of this analysis, the highest score was 5 as we did not perform the *Process* and *Outcome* evaluations.

#### Inter-rater Reliability

Inter-rater reliability was achieved through comparing two separate evaluations by different raters. Regarding inter-rater reliability for the application of EquiFrame to the Malawi National HIV and AIDS Policy, there was a 100% agreement on the scores regarding ‘Core Concept Quality’ for the policy. However, for ‘Core Concept Coverage,’ there was 70% agreement for Core Concepts identified by the raters in some sections of the policy. For example, in Chapter 1 (1.3.5) of the policy relating to ‘Sectoral Policies,’ the policy states that ‘This will in-turn help prevent the further spread of HIV infection, promote access to treatment, reduce stigma and discrimination, protection and empowerment of the key and vulnerable populations, gender inequalities and mitigate health, socio-economic and psycho-social impact of HIV and AIDS and fulfilment of human rights and freedoms.’ For this section, the Core Concepts of *Coordination of Services, Prevention, Access, Non-discrimination* and* Protection from Harm* were identified by both raters. However, one rater also identified the Core Concept of *Participation*. Similarly, in Chapter 2 (2.3) (iv) of the policy relating to ‘Policy Outcomes,’ it states that ‘Improved legal, regulatory enabling environment, evidence based planning, management and coordination of HIV and AIDS interventions.’ For this section, *Coordination of Services* and* Accountability* were identified by both raters as Core Concepts, while the Core Concept of *Quality* was also identified by one rater. Differences in the identification of Core Concepts in each section of the policy was resolved through discussion by the two raters to achieve agreement.

The EquIPP analysis of the review process for developing a revised Malawi National HIV and AIDS Policy indicated that in terms of inter-rater reliability, there was 94% agreement on the scores regarding key actions during the process. Accordingly, out of 17 Key Actions, there was disagreement in assessing the evidence in support of one of these and this will be discussed later.

## Results

### EquiFrame Analysis of Policy Content

Table illustrates the scorings for *EquiFrame* for the policy. The policy scored above 50% on each of *EquiFrame’s* summary indices. Accordingly, the policy received an ‘Overall Summary Ranking’ of ‘High.’

**Table:** Scoring of the Malawi National HIV and AIDS Policy on EquiFrame’s Summary Indices

	**Vulnerable Group Coverage (%)**	**Core Concept Coverage (%)**	**Core concept Quality (%)**	**Overall Summary Ranking**
Malawi National HIV & AIDS Policy	66.7	81	100	High

#### Core Concept Coverage

The policy scored 81% for ‘Core Concept Coverage.’ The policy explicitly mentioned the Core Concepts of *Coordination of Services* and *Quality* most frequently at 48 and 34 times, respectively. Most frequently mentioned Core Concepts also included *Accountability*, *Cultural Responsiveness* and *Participation*, mentioned 29, 26 and 22 times, respectively. The Core Concepts of *Privacy*, *Autonomy* and *Individualized Services* were mentioned less than three times in the policy. Noticeably not mentioned in the policy were the Core Concepts of *Entitlement*, *Capability Based Services*, *Family Resource* and *Family Support*. For a detailed summary of all Core Concepts of human rights and the key words used in the policy, please see Appendices 1 and 5.

#### Vulnerable Group Coverage

The policy mentioned 8 of the 12 Vulnerable Groups defined in EquiFrame. This represents a 66.7% coverage of Vulnerable Groups in the policy. The Vulnerable Groups of people *Suffering from Chronic Illness* (specifically people living with HIV) and people with an *Increased Relative Risk for Morbidity* were mentioned most frequently, both mentioned 15 times in the policy. The Vulnerable Group of *Women Headed Households* was also mentioned frequently, approximately 14 times. Four Vulnerable Groups that were not mentioned in the policy were the *Aged*, *Ethnic Minorities*, those *Living Away from Services* and *Displaced Populations*. ‘Vulnerable Group Coverage’ for the policy is outlined in Appendix 2.

Notably, the policy mentioned 5 other vulnerable groups that are not included in EquiFrame, including drug users, prisoners, girls and key populations (referring to sex workers and MSM). The policy also mentioned ‘*vulnerable populations’* 18 times, although these were not defined.

#### Core Concept Quality

Chapter 1 of the policy states the intention to monitor and evaluate the 8 thematic priorities outlined within the policy. The policy states that there will be a separate implementation plan and a monitoring and evaluation strategy to operationalize the policy. The Malawi National HIV and AIDS Strategic Plan, under the ‘Legal and Policy Environment*’* section, states that the plan is there to operationalize the National HIV and AIDS Policy. Similarly, in the Malawi National HIV and AIDS Monitoring and Evaluation Plan, it is clearly stated that it was developed to measure the performance of the execution and implementation of the Malawi National HIV and AIDS Strategic Plan.^21^ Both the strategic plan and the monitoring and evaluation plan have 8 thematic areas as outlined in the policy. As a specification is explicitly outlined to monitor and evaluate the Core Concepts of human rights included in the policy through the strategic plan, all of the Core Concepts were rated highly with a score of 4 in terms of quality of commitment to the Concepts. Consequently, the quality of the Core Concepts in the policy was scored as 100%.

### EquIPP Analysis of the Policy Review Process

Figure illustrates the scorings for the policy process on each of the 17 Key Actions as defined by EquIPP. Sixty-five percent of the Key Actions received a rating of above 4. This indicates that there is sufficient evidence to suggest that the review process took reasonable steps to promote participation and engagement of vulnerable groups through these actions. Thirty-five percent of the Key Actions were given a score of less than 4 because there was insufficient evidence that the action was undertaken, it may have been considered but incomplete or at times there was only partial engagement with Key Actions.

**Figure:**
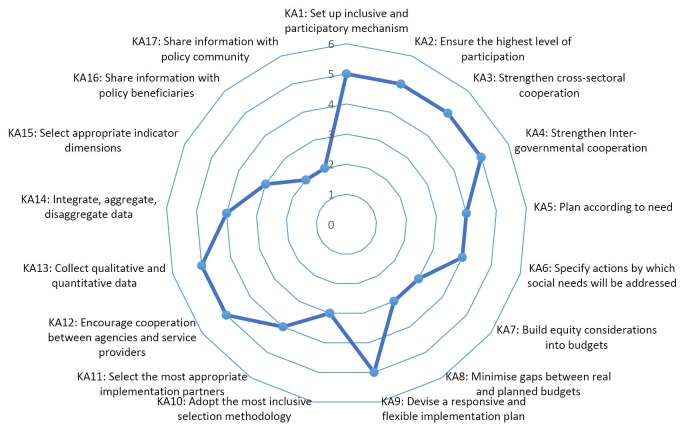
Summary of EquIPP Scores for the Inclusiveness of the Review Process of the Malawi National HIV and AIDS Policy.

An in-depth analysis of the results based on evidence from the interviews and the documentation revealed that there were no significant differences in the scores between both sources of data for the majority of key actions. In 10 of the key actions, the policy scored the same regardless of whether it was an interview or information from documentation, while six of the key actions had a difference of 1 in their scores. However, in an isolated case, one key action had a difference of 2 between the scores derived from each of these sources. It should be noted that the differences were a result of inconsistent evidence. It was noted that throughout the interviews, the respondents were quick to brand the process as inclusive and participatory without necessarily having concrete evidence to support such an assertion. The rating of information from documentation often gave more reliable evidence than the interviews because the former was easier to verify. The comparison of the two methods of rating the policy (interviews and documentation) contributed to the analysis process being more comprehensive and robust.

## Discussion

### EquiFrame

The policy scored an ‘Overall Summary Ranking’ of ‘High’ on EquiFrame’s summary indices, covering 67% of Vulnerable Groups, and 81% of Core Concepts with a ‘Core Concept Quality’ score of 100%. In terms of ‘Vulnerable Groups Coverage,’ the policy also included additional groups such as MSM, sex workers and prisoners, who are not mentioned in EquiFrame. The policy can therefore be considered to be more inclusive than the score of 67% at present suggests. Indeed, if the above 5 mentioned groups were added to the 12 groups included in EquiFrame, it would mean that the policy referred to 13 of 17 groups; for whom there is evidence of their marginalization. This would equate to a coverage of over 70%.

The policy also explicitly mentioned an extensive range of Core Concepts of human rights that include civil, political, economic, social and cultural rights. Chapter 3 of the policy priority area 5 encompasses the Core Concepts of *Non-discrimination*, *Privacy*, *Protection from Harm*, *Participation*, *Liberty* and *Cultural Responsiveness*. The policy has committed to ensure that participation, protection and empowerment of individuals in the HIV and AIDS context are advanced. Nevertheless, the policy fails to recognize HIV and AIDS as conditions that necessitate family resources and support. The policy does not explicitly mention the need for those affected to have support from family as a core concept of human rights. Provision of health services should be directed and centered at the family, especially for persons with disabilities, so that the individuals can benefit from the unity, integrity, capacity and quality of life provided by family.^24^ Similarly, those affected by HIV also need to be supported by their families materially and psychosocially. This is an obvious means of eliminating stigma and discrimination against people who are affected by HIV and AIDS.

Consideration of all the omissions in the policy is beyond the scope of this discussion, but we do want to illustrate some. For instance, less mention is given to persons with disabilities in the proposed interventions mentioned in the policy. People with disabilities need to be considered in all aspects of policy processes in order for their needs to be considered. The International Labor Organization,^25^ emphasizes the need to include people with disabilities in interventions and strategies intended to reduce poverty, since in most of these strategies people with disabilities are not explicitly targeted. The UN Economic and Social Council^26^ emphasizes the need of governments and private institutions providing health services to critically observe the principle of non-discrimination of persons with disabilities.^27^ HIV/AIDS and disability are closely interlaced. According to the United Nations, persons with disabilities are at increased risk of exposure to HIV; moreover, persons with HIV/AIDS are at risk of developing a disability on a permanent or episodic basis as a result of their condition.^28^ Therefore, the policy should explicitly and comprehensively address people with disabilities, especially in relation to informational, financial and physical access to HIV related services.

Vulnerability is not a closed category, but rather a fluid and interacting set of contextual and individual factors. The policy ought to consider how interventions could reach those living in hard to reach areas, however, our analysis indicates that it has failed to consider those living away from health services. The lack of accessible transportation and expense of transportation among a group who are frequently disproportionately poor, may for instance restrict the ability of persons with disabilities to return for health tests results, compared to their peers without disabilities^29^ highlighting the interaction of different types of vulnerable contexts.

The implementation of the policy has hinged on a multi-sectoral approach with one coordinating body. Chapter 5 of the policy outlines the need for coordination and provision of quality services in order to achieve the goal of the document. It is noteworthy that *Coordination of Services*, *Participation* of people, *Accountability* and *Access* to services that are of good *Quality* have been addressed throughout the policy. In relation to *Coordination of Services*, the policy states that ‘the country shall facilitate linkages among sectors for an effective collaboration and networking for a coordinated response to HIV and AIDS through sectoral integration, alignment and mainstreaming of HIV and AIDS in policies, programmes and budgets’ (p. 14). As Mendizabal et al argue, the needs of vulnerable individuals and groups require a simultaneous and integrated approach fostered with the cooperation of implementers at all levels among civil society organizations and non-governmental organizations in order to avoid duplication or ad-hoc service provision.^30^

## EquIPP

The analysis of the policy revision process indicated that reasonable steps were considered to involve vulnerable groups either directly or through their representative organizations in the planning as well as in the implementation of the policy. The final policy document explicitly reflects the involvement of vulnerable groups and consideration of their specific needs. The analysis of the policy revision process therefore established the process to be inclusive and participatory by involvement of the vulnerable groups and their representative organizations. The involvement and participation of vulnerable groups is further recognized in the implementation, monitoring and evaluation of the policy. However, evidence gathered from the majority of interview respondents and policy process documentation indicated that the final revised policy document lacked proper dissemination to the actual beneficiaries and there were insufficient feedback loops to the community during the revision process.

An inclusive policy is a direct outcome of a meaningful participatory policy procedure. The creation of participatory mechanisms that directly engage marginalized groups of people provide them with a forum to influence the overall policy outcome.^13,18^ The Malawi HIV and AIDS Policy has included most of the vulnerable groups defined in EquiFrame. The revision process further included and considered the rights of other groups not mentioned in EquiFrame. The policy mentions prisoners and other populations living in closed settings, who the society consider as criminals and offenders. Frequently, the marginalization of vulnerable groups is overlooked by countries in the planning and implementation when developing strategic documents for the betterment of vulnerable groups.

People in authority, especially politicians, may consider targeting those who already have better access to health services as a cheaper and easier way to achieve short-term health gains, while neglecting the needs of those who are harder to reach, who may in fact need the services most.^10^ However, the Malawian policy interestingly considers MSM and sex workers, despite the legal environment not being favorable to these groups. The Malawi penal code criminalizes the practice of sodomy and homosexuality as well as sex work. However in 2012, the Malawi President called on the National Assembly to repeal the existing sodomy law under which same-sex relations are punishable by up to 14 years in prison. Following some resistance to the call to repeal, the President issued a moratorium on the enforcement of the sodomy law. The moratorium is currently being challenged in courts. Our analysis revealed that the groups consulted in the policy process included female sex workers, MSM, prisoners, people with disabilities, women and people living with HIV. MSM were consulted and included in the review process - despite the oppressive legal environment - to the same extent as the other mentioned groups. The country’s implementation plan for the policy has indicated and suggested how the intervention must take into account the different situations of different target groups; including MSM. Despite the legal environment MSM were engaged fully in the policy process and this was facilitated through a specific organization; the Centre for the Development of People (CEDEP); which states its aims to protect the rights of sexual minority groups (sex workers, LGBT, prisoners) and the organization is one of the key stakeholders in the Malawi’s National HIV and AIDS response. The embracing of MSM issues by this organization represents an interesting and promising way in which State law can be challenged through established and respected organisations embracing issues that are outside what is ‘allowed’ in conventional government discourse. The Malawi Policy on HIV and AIDS demonstrates policy agility by incorporating the reality of what in this country is considered illegal activity, into the needs for adequate service provision for that group. In doing so, it is diminishing the marginalization of such groups.

The policy revision process and the development of its implementation, monitoring and evaluation plans appear to have taken relevant and key stakeholders on board. The Core Concepts of *Coordination of Services* and *Quality* of services can never be achieved without the existence of strong cross-sectoral implementation of programmes. Governments should know that the promotion of social inclusion and equity at an organizational or institutional level is not only about changing traditions or behavior, but also changing the values and norms that influence how government officials perceive and understand social inclusion.^14^ It is very important to note that an effective change can only be achieved with the meaningful involvement of a variety of stakeholders in every step of the policy process. Malawi is moving towards achieving a strengthened cross-sectoral and intergovernmental cooperation in its fight against HIV and AIDS. The country involved all of its key ministries including Health, Gender, Agriculture, Education, and Local Government in the policy review process. This is significant for the country, which has acknowledged that HIV and AIDS issues are cross-cutting and necessitate a collaborative approach.

Sharing information with policy beneficiaries and communities is pivotal in achieving the desired outcomes of the policy. Limited access to relevant policy information prevents people from fully participating in the issues that affect them in their societies; and is categorized as an indicator and driver of the social exclusion of such vulnerable groups.^31^ The findings of this study reveal that the revision process lacked a comprehensive and inclusive dissemination system. The fact that the policy was disseminated at higher levels only indicates that the process did little to share the policy information with beneficiaries and communities. The failure to trickle down the policy dissemination to communities has been attributed to a lack of financial resources. However, this lessens the efforts the country is taking to end the epidemic. We suggest that the dissemination could have used existing community structures to disseminate the policy information more effectively. Although the policy was translated into the main local language, it was never printed in hard copies, thereby making it difficult for the policy beneficiaries to access and benefit from the policy. It was also not made available in other accessible formats. This affirms that many people will not be aware of their existence and entitlements under this policy.

Lack of policy information for beneficiaries constitutes a major barrier to access services associated with the policy,^32^ It is of great importance to acknowledge that information is a public good and should be accessed by all freely without hindrances.^31^ For the vulnerable groups and affected individuals to participate and engage fully with policy processes, government must therefore provide them with relevant and necessary policy information. The information sharing gap has not only been observed with regards to policy dissemination, but also in the government’s failure to explicitly isolate HIV and AIDS expenditures in different sectors. The Government financial reports do not outline specific expenditures on HIV and AIDS in each sector, and instead the report provides a general overview of institutions’ expenditure. It is the government’s responsibility to produce budget and financial reports that are non-technical and easy to understand by policy beneficiaries. Governments must thus ensure that any communication relating to the policy and its benefits is disseminated in plain language and accessible to all.

The legitimate inclusion of vulnerable groups in policy documents extends beyond participation in the policy process. Inclusion involves continuous creation of a community in a coproducing process, of policies and programmes that define and address public issues.^14^ Equally, monitoring and evaluation frameworks should be designed in a participatory manner to allow the capturing of experiences and impact the policies have on vulnerable groups.^14^ Participation of vulnerable groups in policy processes contributes to documents that aim to deliver better public services. The Civil Society Organizations representing vulnerable populations participated in different forums, at the Parliamentary and Cabinet levels. The vulnerable groups and their representatives were involved throughout the process. Their involvement in the drafting process of the policy is reflected in the final document where their interests, or those of their constituencies, are explicitly presented. For example, the policy has a specific section on those living with HIV regardless of gender or sex. The policy-makes it clear that the country is gearing at scaling up preventions services especially to the key populations affected - female sex workers and MSM.

The Malawi National HIV and AIDS Policy, its implementation plan and monitoring and evaluation plan form a strong complementarity. The implementation plan provides the relevant information and interventions that address the proposed 8 priority areas of the policy. The monitoring and evaluation plan is comprehensive and has well-defined indicators that are simple to understand by all types of beneficiaries. The simplicity of the indicators enhances an effective monitoring of the performance of policy implementation by the public.

Of note, the policy underwent a similar process of content analysis through the application of EquiFrame by researchers in 2012. The outcome of the analysis was that the policy was scored an ‘Overall Summary Ranking’ of ‘Moderate.’^8^ The policy scored low for the quality of the Core Concepts as the policy stated an intention to monitor and evaluate Core Concepts without any specific policy action to address the Concepts. However, in this analysis, the revised policy has scored an ‘Overall Summary Ranking’ of ‘High.’ This score is as a result of the strengthened intention to monitor and evaluate the proposed Core Concepts through development of the monitoring and evaluation plan. In both evaluations, the policy received the same scores for coverage of Core Concepts of human rights and inclusion of Vulnerable Groups of 81% and 66.7% respectively.

The current analysis met the challenge of respondents not being able to recall the policy processes in detail. Some respondents found it difficult to recall the actual policy processes, attributing it to the fact that the research was conducted sometime after the revision was concluded. Some respondents therefore opted to answer selectively based on what they remembered.

Based on the findings above and our experience in conducting this research, we conclude with a number of recommendations:

1. There is need for EquiFrame to be used flexibly, particularly regarding additional vulnerable groups relevant to the context of the research; and possible omitting some of the vulnerable groups covered by EquiFrame, if there is evidence to support their irrelevance to the specific policy area of concern. Thus, groups such as sex workers, MSM, prisoners and many others may be considered as vulnerable in a range of different contexts. However, we want to acknowledge that vulnerability is not an attribute but an experience; arising from how some groups are positioned by social attitudes and structures that disadvantage them.^33^
2. It is recommended that policy should explicitly consider and explicitly resource dissemination of information to beneficiaries at community level; recognizing that policy information is a public good, hence the need to share it with the public at all times.
3. There is value in combining different types of evidence to ascertain the veracity of inclusion in the policy process. Here we used both interviews with stakeholders, including the Policy-makers themselves, and supporting documentation.
4. The measurement of inclusion in terms of both its process and content allows for a more comprehensive analysis of the likely benefits of policy to marginalized groups. Such measurement provides a target for improvement where the policy process or content has been found to be weak. Combining EquiFrame and EquIPP is one way of doing this.
5. In the case of the Malawian HIV and AIDS Policy, our analysis indicates that the revision process took reasonable steps to engage the participation of vulnerable and marginalized groups throughout planning, implementation, monitoring and evaluation phases. The results further reveal that the final revised policy document covered Core Concepts of human rights and included many Vulnerable Groups. We have highlighted how these positive attributes of the policy can be further strengthened both in terms of policy process and policy content. Of particular concern is that the revised policy has only been disseminated to beneficiaries at national level only. This lack of dissemination to beneficiaries at local levels falls short of the commitment to social inclusion apparent in much of the policy. This particular short-coming should now be addressed through a systematic campaign of dissemination at community level.
6. A significant challenge remains in identifying the most appropriate ways in which States can be supported in ‘domesticating’^34^ international human rights treaties and conventions. Not all governments accept the universality of human rights, and even where they do, United Nations’ conventions may make provision for the “progressive realization”^35^ of such rights, generally based on the resources available within a country. Liberal and inclusive attitudes, and the strength and influence of civil society are an important resource in this regard, and a lack of them, is also a resource challenge that can influence the realization of rights. How then to persuade reluctant governments to be more inclusive of marginalized groups – particularly when their democratic power-base may rely on pandering to prejudice, stigmatizing and ‘othering’ – is at the crux of the broader adoption of basic human rights principles. Here we have sought to highlight and encourage good policy practices that represent progress towards stronger and more inclusive human rights in Malawi; seeing this as a process, not an outcome. However, this is not the only way to progress human rights; and we recognize that other approaches; such as strategic litigation, public protest and human rights advocacy highlighting the failures of government, are equally legitimate, and in our view, in no way incompatible with the approach described in this paper.

## Ethical issues

Ethical permission was obtained from the National Health Sciences Research Committee in Malawi and from the Health Policy and Management and Centre for Global Health Research Ethics Committee at Trinity College Dublin, Dublin, Ireland.

## Competing interests

Authors declare that they have no competing interests.

## Authors’ contributions

Conception and design: MJC; Acquisition of data: MJC and SG; Analysis and interpretation of data: MJC; Drafting of the manuscript: MJC; Critical revision of the manuscript for important intellectual content: JM and TH; Supervision: MM.

## Authors’ affiliations

^1^Centre for Global Health, Trinity College Dublin, Dublin, Ireland. ^2^Department of Nutrition, HIV and AIDS, Lilongwe, Malawi. ^3^Centre for Global Health & School of Psychology, Trinity College Dublin, Dublin, Ireland. ^4^Centre for Rehabilitation Studies, Stellenbosch University, Stellenbosch, South Africa. ^5^Olomouc University Social Health Institute, Palacky University, Olomouc, Czech Republic.

## Key messages

**Implications for policy makers**

Policy-makers can benefit from the results of our study in the following ways:

- Policy-makers can use EquiFrame flexibly, particularly regarding additional vulnerable groups relevant to the context of the research; and possible omitting some of the vulnerable groups covered by EquiFrame, if there is evidence to support their irrelevance to the specific policy area of concern. Thus, groups such as sex workers, men who have sex with men (MSM), prisoners and many others may be considered as vulnerable in a range of different contexts. However, we want to acknowledge that vulnerability is not an attribute but an experience arising from how some groups are positioned by social attitudes and structures that disadvantage them.
- Policy-makers will be able to develop policies that explicitly consider dissemination of information to beneficiaries at community level considering that policy information is a public good, hence the need to share it with the public at all times.
- The measurement of inclusion in terms of both its content and process allows for a more comprehensive analysis of the likely benefits of policy to marginalized groups. Such measurement provides a target for improvement where the policy content or process has been found to be weak. Combining EquiFrame and EquIPP is one way of doing this.

**Implications for public**

The legitimate inclusion of vulnerable groups in policy documents extends beyond participation in the policy process. Inclusion involves continuous creation of a community in a coproducing process of policies and programmes that define and address public issues. It is essential that the public actively participate throughout planning, implementation, monitoring and evaluation phases of policies. Monitoring and Evaluation frameworks should be designed in a participatory manner to allow the capturing of experiences and impact the policies have on vulnerable groups. Lack of policy information for beneficiaries constitutes a major barrier to access services associated with the policy. For vulnerable groups and affected individuals to participate and engage fully with policy processes, government must therefore provide them with relevant and necessary policy information.

## Appendix 1

**Table:** EquiFrame’s Core Concepts of Human Rights, and Key Words Used in the Malawi National HIV and AIDS Policy

**No.**	**Core Concept**	**Frequency**	**Quality**	**Key Words for Core Concepts of Human Rights in the Policy**
1	Non Discrimination	14	4	States the prohibition of discrimination on the basis of HIV status, race, sexual orientation and culture
2	Individualized services	1	4	Institutions shall facilitate creation of an enabling environment to reduce individual and societal vulnerability to HIV and AIDS
3	Entitlement	0	0	
4	Capability-based services	0	0	
5	Participation	22	4	This is aimed at ensuring protection, participation and empowerment of individuals in the context of HIV and AIDS
6	Coordination of services	48	4	The country shall facilitate linkages amongst sectors for effective collaboration and networking for coordinated response to HIV and AIDS through sectoral integration, alignment and mainstreaming of HIV and AIDS in their policies, programmes, strategic plans and budgets
7	Protection from harm	15	4	The policy states that all persons are equal before and under the law and are entitled without any discrimination to the equal protection and equal benefits. Every person shall have access to public institutions for the protection when rights have been violated
8	Liberty	4	4	The Policy states that every person shall enjoy the rights and liberties guaranteed by the Constitution legislations and international instruments
9	Autonomy	1	4	The Policy shall respect individual autonomy including freedom of expression as provided in the Constitution
10	Privacy	2	4	Every person shall enjoy right to privacy as provided in the Constitution
11	Integration	6	4	All sectors shall effectively integrate HIV and AIDS in their policies, programmes, strategic plans and budgets
12	Contribution	11	4	The policy will put deliberate efforts to make sure individuals affected by HIV and AIDS contribute in the economic development of the country
13	Family resource	0	0	
14	Family support	0	0	
15	Cultural responsiveness	6	4	The policy recognizes that every person has freedom to participate in culture of choice regardless of HIV status. The policy further promote access to public institutions for protection when rights of affected people are violated
16	Accountability	29	4	The policy fosters accountability by ensuring that strategic information for planning and decision making for the HIV Response is generated in a collaborative manner. In addition, the policy shall be reviewed regularly
17	Prevention	26	4	The policy states that it shall guide in the implementation of programmes aimed at providing quality HIV and AIDS services to prevent further spread of the virus
18	Capacity building	15	4	In order to enhance efficient and effective implementation of the National Response, the policy shall ensure that the Capacity Development Plan is implemented
19	Access Total	28	4	
	*General*	11	4	The policy shall ensure universal access to HIV related services to all
	*Economic*	9	4	The policy states that it shall promote economic development interventions focusing on the socio-economic impact of people affected by HIV and AIDS at all levels
	*Physical*	0	0	
	*Informational*	8	4	The policy states that all affected individuals will access to information regarding their health as accorded in the Constitution under Chapters III and IV
20	Quality	34	4	The policy states that it shall guides implementation of programmes towards provision of quality health services to people living with HIV, their dependents and communities
21	Efficiency	5	4	In order for the policy to achieve efficient and effective implementation of the HIV National Response, it shall ensure the Capacity Development Plan is implemented

## Appendix 2

**Table:** EquiFrame’s Vulnerable Groups Mentioned in the Malawi National HIV and AIDS Policy

**No.**	**Vulnerable Group**	**Frequency**
1	Limited resources	9
2	Increased relative risk for morbidity	15
3	Mother/child mortality	6
4	Woman-headed household	11
5	Children (with special needs)	7
6	Aged	0
7	Youth	5
8	Ethnic minorities	0
9	Living away from services	0
10	Displaced populations	0
11	Suffering from chronic illness (People living with HIV)	15
12	Disabled	3

## Appendix 3

**Table:** Interview Participants

**Sector**	**Number**
Public (Government)	4
Civil Society	4
Development Partners	2

## Appendix 4

Sources of Documented Evidence

1. Cabinet Paper Report, 2013
2. Guidelines on the utilization of at least 2% ORT budget allocation
3. Issues Paper presented to Cabinet, May 2010
4. Malawi HIV and AIDS Communication strategy, 2012
5. Malawi National HIV and AIDS Monitoring and Evaluation Plan, 2015-2020
6. Malawi National HIV and AIDS Policy, 2011-2016
7. Malawi Partnership Forum Terms of References and Guidelines
8. National AIDS Commission and Government Financial papers, 2014
9. National AIDS Commission Data Quality Audit reports
10. National AIDS Commission Monitoring and Supervision reports
11. National HIV and AIDS Strategic Plan (NSP), 2015-2020
12. Policy Report, 2010
13. Proposed Policy Framework for policy review, 2010
14. Report on Consultations with Communities, 2010
15. Report on Consultations with Members of Parliament
16. Terms of References for Nutrition, HIV and AIDS Steering Committee, 2010
17. Terms of References for Review Process Facilitators, 2010
18. Terms of References for Technical Working Groups, 2010

## Appendix 5

**Table:** EquiFrame’s Core Concepts Coverage for the Malawi National HIV and AIDS Policy

**No.**	**Core Concept/Principle**	**Frequency**	**Coverage**
**1**	Constitutional (5 Core Concepts)	5	100%
**2**	Ethical (8 Core Concepts)	6	75%
**3**	Administrative (8 Core Concepts)	6	75%
